# Current View of Diagnosing Small Fiber Neuropathy

**DOI:** 10.3233/JND-200490

**Published:** 2021-03-02

**Authors:** Lisette R.M. Raasing, Oscar J.M. Vogels, Marcel Veltkamp, Christiaan F.P. van Swol, Jan C. Grutters

**Affiliations:** a ILD Center of Excellence, Department of Pulmonology,St Antonius Hospital, CM, Nieuwegein, The Netherlands; b Department of Neurology, St Antonius Hospital, CM, Nieuwegein, The Netherlands; c Department of Clinical Physics, St Antonius Hospital, CM, Nieuwegein, The Netherlands; d Division of Heart and Lungs, University Medical Center Utrecht, CX, Utrecht, The Netherlands

**Keywords:** Autonomic dysfunction, nerve fiber density, small fiber neuropathy, diagnostic accuracy

## Abstract

Small fiber neuropathy (SFN) is a disorder of the small myelinated A*δ*-fibers and unmyelinated C-fibers [5, 6]. SFN might affect small sensory fibers, autonomic fibers or both, resulting in sensory changes, autonomic dysfunction or combined symptoms [7]. As a consequence, the symptoms are potentially numerous and have a large impact on quality of life [8]. Since diagnostic methods for SFN are numerous and its pathophysiology complex, this extensive review focusses on categorizing all aspects of SFN as disease and its diagnosis. In this review, sensitivity in combination with specificity of different diagnostic methods are described using the areas under the curve. In the end, a diagnostic work-flow is suggested based on different phenotypes of SFN.

## BACKGROUND

### Etiology

Small fiber neuropathy (SFN) is a disorder of the small myelinated A*δ*-fibers and unmyelinated C-fibers [5]. Incidence and prevalence are estimated to be 12/100.000 and 53/100.000 respectively and are expected to rise with increasing awareness of SFN worldwide [6]. SFN can affect either small sensory fibers, autonomic fibers or both, resulting in sensory changes, autonomic dysfunction or combined symptoms [7]. As a consequence, the potential symptoms are numerous and show a large impact on quality of life [8]. General symptoms are fatigue, cognitive disturbances, widespread musculoskeletal pain, hea-dache and temporomandibular disorder [9, 10]. Som-atic small nerve fibers transmit information about temperature, pain and itch [9, 11, 12]. The autono-mic small nerve fibers are responsible for thermoregulatory, sudomotor, cardiovascular, gastrointestinal, urogenital and other autonomic functions [7, 9]. SFN is associated with a great variety of diseases as underlying mechanisms, but can also present idiopathic [5]. Table 1 shows an overview of some underlying disorders [5, 9]. Common nerve conduction tests only assess large myelinated nerve fibers. As a consequence, SFN is difficult to diagnose following the regular procedures [11, 12]. Currently, the prevalence of SFN is probably highly underestimated due to lack of a gold standard and awareness among clinical physicians [9]. Improving diagnostic methods is important to improve recognition of symptoms in SFN patients, it can improve insight of pathophysiology and will facilitate future drug trials.

**Table 1:** Underlying diseases associated with SFN

Associated diseases of small fiber neuropathy [9, 125]
•Idiopathic
Hereditary
•Fabry’s disease [126]
•Mutation in sodium channels [1]
•Wilsons disease [127]
•Familial amyloidosis [128]
Metabolic
•Diabetes mellitus [67]
•Impaired glucose intolerance [24]
•Vitamin B12 deficiency [129]
•Copper deficiency [130]
•Abnormal thyroid function [131]
Infectious
•HIV [132]
•Lyme [133]
•Hepatitis C [134]
Toxic
•Alcohol [135]
•Chemotherapy [136]
•Neurotoxic drugs [32]
•Vaccine-associated [134, 137]
Immune-mediated
•Fibromyalgia [138]
•Monoclonal gammopathy [139]
•Ehlers-Danlos [140]
•Sarcoidosis [141]
•Rheumatic diseases (undifferentiated connective tissue disorders, rheumatoid arthritis, psoriasic arthropathy) [32]
•Sjögren syndrome [142] Primary systemic amyloidosis [139]
•Acute inflammatory small fiber neuropathy [32]
•Lupus [144]
•Connective tissue disease [32]
•Chronic inflammatory demyelinating polyneuropathy [145]

### Pathophysiology

**Fig. 1:**
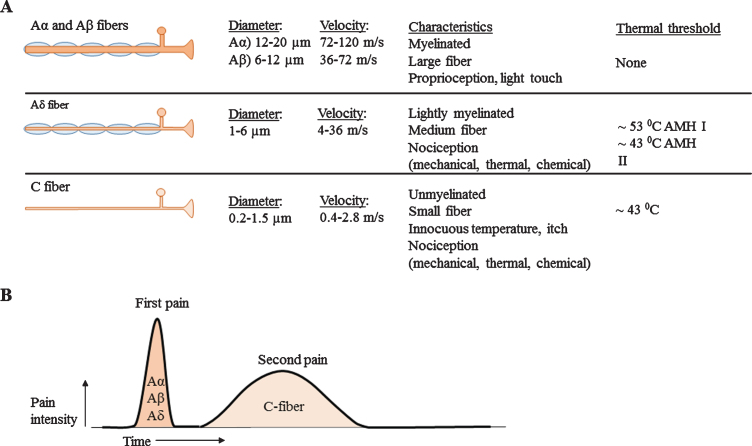
A) Overview of nerve fiber sizes, conduction velocities and other characteristics. A*α* and Aβ fibers are large and myelinated nerve fibers, A*δ* nerve fibers are small myelinated nerve fibers and C-fibers are small unmyelinated nerve fibers. B) Corresponding pain response. Large nerve fibers show a fast response with high amplitude. The smaller the nerve fiber, the lower the amplitude and the slower conduction velocities.

The peripheral nervous system is classified into different types of nerves, based on diameter, myelin sheet and conduction velocity, see Fig. 1A [11, 13]. A*α*- and Aβ-fibers are classified as large nerve fibers and A*δ*- and C-fibers are classified as small nerve fibers. Small myelinated A*δ*-fibers show faster conduction velocities (4–36 m/s) [14, 15] compared to unmyelinated C-fibers (0.4–2.8 m/s) [15–17], due to larger diameter and myelin [18]. SFN is described as dysfunction of the small nerve fibers. The exact path-ophysiology of isolated SFN is unknown. However, since demyelinating processes do not solely affect small nerve fibers, it is unlikely that this would be the underlying pathogenesis. Distal axonal loss or perhaps extraordinarily neuronal degeneration are therefore more likely to be the underlying cause of SFN [7]. Five decades ago, four stages of neuropa-thy pathology in unmyelinated nerve fibers were def-ined [19]. 1) Mild proliferation: This stage is chara-cterized by merely an increase in number of isolated, small Schwann cell projections. As consequence, these Schwann cells show a more irregular shape. 2) Fiber loss: in a more advanced stage, a decreased amount of fibers in combination with in-creased amount of empty Schwann cells are established. 3) Regeneration: Subsequently, regeneration of unmyelinated fibers associated with signs of fiber loss have been identified. An increment can be noted from the total number of unmyelinated fibers as well as small fibers with a diameter below 0.8μm and empty Schwann cell sub-units. 4) Advanced regeneration: Finally, the amount of empty Schwann cells will return to a normal level. During this stage, only an increase of small nerve fibers with a diameter below 0.8μm, and of small isolated pro-jection of Schwann cells can be distinguished [19]. Patients with diabetic-mediated SFN might show a different pathophysiology compared with other und-erlying etiologies. For example, in diabetic patients, axon swelling seen in skin biopsies can predict pro-gression of distal SFN to proximal large fiber or polyneuropathy [20–22]. In contradiction, recent res-earch which included no diabetic patients, claims that SFN is a stable disorder and rarely progresses [23]. Although symptoms typically are length-dependent, resulting in symptoms in distal extremities [10], it also commonly presents with a non-length-dependent character [24]. Non-length dependent SFN is likely to be associated with immune-mediated conditions and it presents more often in women at younger age [25].

### Somatic system

The somatic nerve system is responsible for voluntary muscle control and sensory function. Sensory function, is broadly divided into special senses and general senses. Special senses include olfaction, vi-sion, hearing, balance and taste. General senses are divided into exteroceptors, (present in skin: nociception (pain, temperature, touch, pressure)), interoceptors (present in viscera: mechanical and chemical stimuli) and proprioceptors (present in muscles, joi-nts and tendons: awareness of posture and movement) [26]. SFN mainly results in symptoms caused by damage of the nociceptive system. As a result patients complain about pain, burning, tingling, prickling, shooting pain or numbness. Due to difference in conduction velocity, A*δ*-fibers are responsible for the sharp, pricking or first pain response and C-fibers for the burning or second pain response, see Fig. 1B [13]. A*δ*-fibers which respond to heat, are divided into type I and II A mechano-heat (AMH) units. AMH type I nerve fibers have a high response threshold (>53C), and their discharge rate increase during a prolonged stimulus. Typically type I AMH fibers show a higher sensitivity for mechanical stimuli compared to AMH type II fibers. AMH type II fibers have a short-latency adapting response, they have a lower threshold for heat stimuli (43–47C) and exhibit slower conduction velocities [27]. As consequence, AMH type I fibers are responsible for first pain sensation of mechanical stimuli and AMH type II fibers are involved in first pain sensation of heat pain stimuli [13, 18]. C-fibers can be polymodal; responsive for noxious, thermal and mechanical stimuli. In addition, they can be responsive for a specific stimulus, but also for multiple stimuli or for non-specific stimuli [28]. Table 2 shows an overview of functions from specific small fiber types. Also specific temperature thresholds are shown, which are used for Quantitative Sensory Testing (QST) measurements.

**Table 2:** Overview of different nociception receptors with corresponding small nerve fiber type [15]

Receptor type	Fiber group	Modality
Cutaneous and		Touch
subcutaneous
mechanoreceptors
• Hair down	A*δ*	• Light stroking
Thermal receptors		Temperature
• Cold receptors	A*δ*	• Skin cooling (25^0^C)
• Warm receptors	C	• Skin warming (41^0^C)
• Heat nociceptors	A*δ*	• Hot temperatures (>45^0^C)
• Cold nociceptor	C	• Cold temperatures (<5^0^C)
Nociceptors		Pain
• Mechanical	A*δ*	• Sharp, pricking pain
• Thermal-mechanical	A*δ*	• Burning pain
• Thermal-mechanical	C	• Freezing pain
• Polymodal	C	• Slow, burning pain
Muscle and skeletal		Limb proprioception
mechanoreceptors
• Stretch-sensitive	A*δ*	• Excess stretch or force
free endings

### Autonomic system

**Fig. 2:**
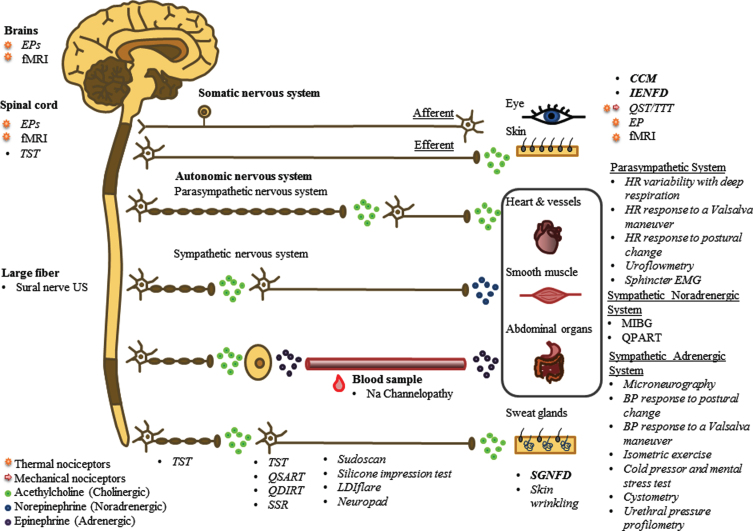
Complete overview of the nervous system, showing anatomical differences between the somatic and autonomic system and differences in nerve anatomy and use of neurotransmitters. In addition, all diagnostic methods are presented at their corresponding measuring area. Different font styles are used to discriminate between methods based on NFD (bold), small nerve fiber function (italic) and imaging (normal). Moreover, for some functional tests, an additional mark is established to discriminate between tests based on thermal and/or mechanical nociceptors. *Abbreviations:* EPs, evoked potentials; fMRI, functional magnetic resonance imaging; TST, thermoregulatory sweat testing; US, ultrasound; CCM, corneal confocal microscopy; IENFD, intra-epidermal nerve fiber density; QST, quantitative sensory testing; TTT, temperature threshold testing; HR, heartrate; EMG, electromyography; MIBG, ^123^I-meta-iodobenzylguadine; QPART, quantitative pilomotor axon-reflex test; BP, blood pressure; SGNFD, sweat gland nerve fiber density; LDIflare, laser Doppler imaging flare; QSART, quantitative sensory axon reflex test; QDIRT, quantitative direct and indirect reflex test; SSR, sympathetic skin response.

The autonomic system differs from the somatic system in a way that the somatic nervous system is connected with its target organ via one neuron, while the autonomic nervous system consists of two neurons. The autonomic ganglion forms the synaptic connection between the preganglionic and postgan-glionic neuron. The efferent autonomic nervous system can be divided into the sympathetic system (stress response), parasympathetic system (rest response) and enteric nervous system (digestive sys-tem) [26]. Preganglionic fibers are myelinated and use acetylcholine as neurotransmitters. Postgangli-onic nerve fibers are smaller compared to pregangl-ionic fibers, are unmyelinated and use norepinephrine as neurotransmitter. An exception are sweat glands, which use cholinergic nerves (Fig. 2) [26].

### Diagnostic methods

Various methods have been described to diagnose SFN. Diagnostic methods can be categorized into questionnaires, genetic analysis, quantification of small nerve fiber density (NFD), sensory function tests, autonomic function tests and imaging techni-ques to quantify small nerve fibers [29]. Questionnaires are rather subjective and imaging techniques are only at the beginning of investigating their abi-lity to diagnose SFN. NFD quantification of small sensory nerve fibers and functional quantification of sensory and autonomic small nerve fibers have been regularly investigated and compared. It is im-portant to keep in mind that NFD or functional outcomes are very different measures for SFN [30]. Diagnosing SFN remains challenging and a golden standard is not yet available. The presence of at lea-st two abnormal findings at clinical, QST and skin biopsy examination have been suggested as best dia-gnostic criteria for SFN [31, 32]. However, there remains some controversy on this suggestion [33]. Moreover, the clinical utility of skin biopsy is lim-ited by labor intensity, availability in few centers, high costs and impracticality for longitudinal studies. Therefore, another research group suggests the presence of at least two abnormal findings at clinical, QST and Quantitative Sudomotor Axon-Reflex Test (QSART) examinations for a definite diagnosis [34]. Since no single method is sensitive enough to confirm or exclude SFN, a combination of multiple methods seems to be the best alternative. The more abnormal test results, the more secure the diagnosis will be. A recent study investi-gated six different methods and suggested even a combination of four methods (skin biopsy, Electrochemical Skin Conductance (ESC), Laser Evoked Po-tentials (LEP) and QST) for a definite diagnosis [2].

In order to classify SFN, the following definitions are preferably used [3, 4, 35].

1. *Possible SFN*: symptoms or clinical signs of small fiber damage
2. *Probable SFN:* symptoms or clinical signs of small fiber damage and normal sural nerve conduction studies
3. *Definite SFN*: symptoms or clinical signs of SFN-damage, normal sural nerve conduction studies and decreased intra-epidermal nerve fiber density (IENFD) and/or abnormal quantitative sensory testing (QST) thermal thresholds

### Diagnosis

In order to diagnose isolated SFN, the physician should be aware of the diversity of symptoms patients can have. During physical examination, tendon reflexes should be normal, no symptoms of muscle weakness should be present, vibration and pro-prioception can be both, normal and abnormal [36]. QST or skin biopsy are recommended for a definite diagnosis [31, 35]. However, those methods are time consuming and by far not available in every hospital.

## OBJECTIVES

• Clarify which diagnostic methods are available
• Define diagnostic accuracy of each method
• Present a clinical diagnostic work-flow

## METHODS

First, the huge amount of available diagnostic methods will be clarified to show an organized over-view. Next, a systematic search is performed, to develop an overview of diagnostic accuracy (AUC-values) for each method. The results of a systematic literature search between 2000–2019 are presented focusing on the sensitivity and specificity of all diagnostic methods. It is important to state that since no golden standard is available, the AUC-values are relative measures, based on an imperfect standard.

Literature search is performed on 19 August 2019 in PubMed and Embase. Exclusion criteria were:

• Publication date <2000
• Case reports
• Language other than English
• Animal study
• Large fiber neuropathy

**Fig. 3:**
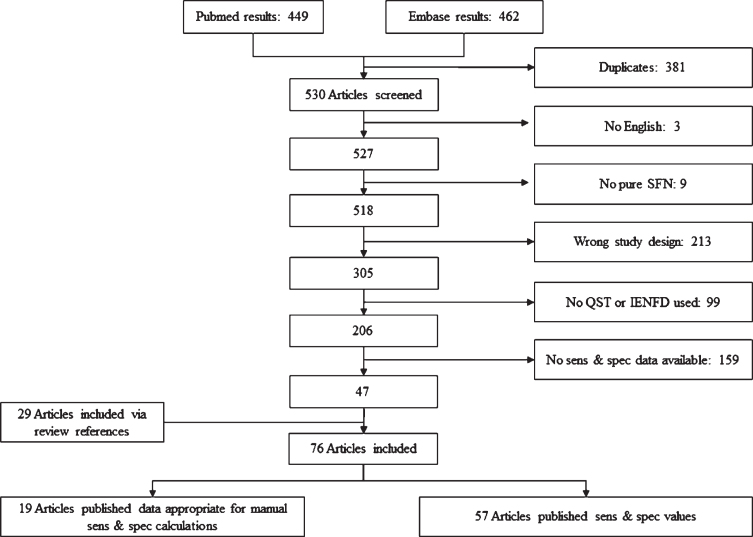
Inclusion results of systematic literature search. Articles excluded with a wrong study design, included review articles, animal models or case reports. If review articles did publish data about sensitivity (sens) and/or specificity (spec), the original papers were looked up. As a result, 29 extra articles were included. 19 articles did publish the test results from all participants, which made it possible to calculate sens & spec. 57 articles did calculate sens & spec based on QST and/or IENFD.

AUC-values are calculated based on published sensitivity and specificity values. Articles were included when sensitivity and specificity were clearly published or were calculated when an overview of test results for all participants was available. The classification of definite SFN is used, in order to determine sensitivity and specificity. Only articles with isolated SFN were included. Figure 3 shows the search results and exclusion criteria. Review articles, animal models and case reports are labeled as “wrong study design”. In the end, several phenotypes of SFN are described based on different symptoms. Depending on the phenotypes, optimal diagnostic methods are suggested.

## RESULTS

### Questionnaires

**Fig. 4:**
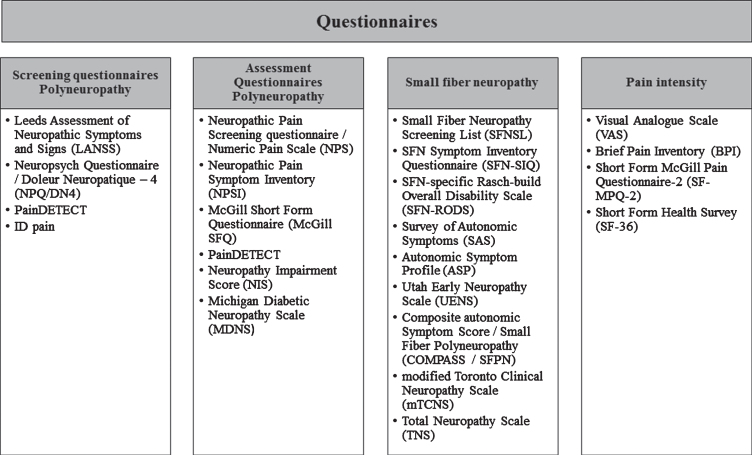
Overview of neuropathy questionnaires, divided into screening questionnaires, assessment questionnaires, specific small fiber questionnaires and pain intensity questionnaires.

Studies focusing on SFN use numerous questionnaires to assess or screen for neuropathic pain symptoms [37, 38]. Neuropathic pain can be caused by small fibers, mixed-fibers or large fibers. The last two causes are classified as polyneuropathy. In order to give a clear overview of the questionnaires, they are categorized into screening questionnaires and assessment questionnaires [37], specific small fiber questionnaires and pain intensity questionnaires, Fig. 4. Screening questionnaires are helpful for easy identification of neuropathic pain, which especially applies for patients with complex medical conditions (e.g., spinal cord injury). Assessment questionnaires are helpful for quantification of neuropathic symptoms [37]. Some questionnaires are listed in two categories, due to multiple types of questions. Which questionnaire will be most suitable depends on the patient population and their symptoms. A good overview of sensitivity and specificity for the screening and assessment questionnaires in polyneuropathies is presented elsewhere [39]. For SFN questionnaires a distinction can be made bet-ween autonomic symptoms (survey of autonomic sy-mptoms (SAS), autonomic symptom profile (ASP)) [40], validation based on Chemotherapy Induced Polyneuropathy (CIPN) (Total Neuropathy Scale (TNS)) [41], validation based on diabetes (modified Toronto Clinical Neuropathy Score (mTCNS) [42], Utah Early Neuropathy Scale (UENS)) [43], pure SFN with the focus on frequency of symptoms (SFN-Symptom Inventory Questionnaire (SFN-SIQ)) [44], isolated SFN with the focus on activity and participation restrictions due to SFN (SFN-RODS) [45] and isolated SFN validated in sarcoidosis patients with the focus on both frequency and intensity of symptoms (Small Fiber Neuropathy Screening List (SFNSL)) [46].

### Sodium channel neuropathy

Voltage-gated sodium channels are responsible for generation and conduction of action potentials in peripheral nerves. Three types of sodium channels are selectively found on nociceptive small fibers. Mutations in Na_v_1.7, Na_v_1.8, Na_v_1.9, encoded by genes *SCN9A, SCN10A* and *SCN11A*, are related with SFN [44, 47, 48]. Approximately 30%of idiopathic SFN patients show a SCN9A mutation [44]. Although a correlation between decreased IENFD and sodium channel mutations exists, [44, 49] IENFD might be normal in patients with Na_v_ mutations and neuropathic small fiber pain symptoms [12, 50]. Na_v_ mutations can result in either gain-of-function or loss-of-function. Gain-of-function mutations of Na_v_1.7 are, amongst others, linked to painful disorders like inherited erythromelalgia (IEM), paroxysmal ex-treme pain disorder (PEPD) and SFN. Na_v_1.7 loss-of-function mutations result into congenital insensitivity to pain (CIP) [51]. Na_v_1.8 and Na_v_1.9 mutations are predominantly linked to peripheral painful neuropathy [1, 52] Na_v_ mutations may also result in other painful diseases, but these are beyond the scope of this review. Table 3 shows the sodium channels and their corresponding type of nerve fibers in which they are present. This discriminates different kind of symptoms as consequence of specific sodium channel mutations. Indeed, not all mutations are likely to be pathogenic, a good overview is published elsewhere [1]. They investigated *SCN9A, SCN10A* and *SCN11A* mutations in 1139 patients with pure SFN. In 11.6%of the participants, over seventy different mutations were found. They advise to consider genetic screening for all patients with pure SFN, independently of clinical presentation or underlying pathology. This way, the number of well-characterized variant of Na_v_ channels will increase. Diagnosing sodium channel neuropathy is not yet part of standard care and serves for additional information. For patients with isolated SFN, knowledge about the origin of their symptoms is of great importance. Research is ongoing for the development of specific sodium channel blockers as treatment.

**Table 3:** Type of sodium channels and corresponding type of neurons

Sensory	Sympathetic	Myenteric
neurons	neurons	neurons
Na_v_1.7	Na_v_1.7	Na_v_1.7
Na_v_1.8
Na_v_1.9		Na_v_1.9

### Quantification of small nerve fiber density

#### Skin biopsy

Protein gene product 9.5 (PGP 9.5) is present in all axons and can be detected with the use of PGP 9.5 antibody. Dermal somatic cholinergic A*δ* and C nerve fibers can be quantified after staining, with the use of microscopy. Morphological changes include swelling, weaker immunoreactivity and change in branching [31]. It is suggested that nerve swelling and excessive proximal branching may be a manifestation of the denervation-reinnervation process in the beginning of the neuropathic process [53]. Most frequently reported pathology in SFN is decreased IENFD. All those changes are also seen in healthy subjects, but to a lower extent [7, 54].

#### Sweat gland nerve fiber density

Sweat glands are innervated by cholinergic sympathetic post-ganglionic C-fibers. Since those lay peripheral, are long, thin and have no myelin, they are prone to nerve damage in many neuropathies. Deteriorated sudomotor function shows to be a reliable predictor for SFN [55, 56]. Sweat gland nerve fiber density (SGNFD) decreases and sweat gland atrophy appears in SFN [57, 58]. SGNFD can be determined with the same biopsy diameter and staining used for IENFD, but requires thicker skin biopsy. Although data confirms validity of assessing SGNFD [57, 58], it is labor intensive (requiring 30–40 hours to evaluate one biopsy) and not suitable for routine use in clinical settings [58].

#### Corneal confocal microscopy

The cornea is highly innervated by A*δ* and C nerve fibers and contains up to 300–400 times more small nerve fibers compared to the skin [59]. Therefore the cornea seems to be the ultimate location for distal SFN to proliferate. Corneal confocal microscopy (CCM) gives the unique possibility to quantify the NFD of the cornea *in vivo*. A correlation between corneal NFD and IENFD has been confirmed. CCM is able to determine the nerve fiber length (NFL), nerve fiber branching (NFB) and tortuosity [60, 61]. Some major advantages are its non-invasiveness, its time-efficiency, its high reproducibility and its allowance for multiple replicates in both cross-sectional and longitudinal studies [60, 61]. Although normative values are available [62] and the technique seems very promising [63], outcomes seem to vary between different research groups and remains a topic of discussion [64]. Automated classification shows representative results [65]. Pitfalls include the small field of view [66] and a lacking consensus for cut off values [67].

### Sensory function tests

#### Quantitative sensory testing (QST)

QST contains a large battery of sensory nerve tests in order to test both large and small sensory fibers [68]. QST involves thermal, pressure, vibration and electrical stimulation. The full QST battery assesses thirteen parameters within seven test procedures [69]. Since the full QST test is time consuming, thermal threshold testing (TTT) is often selected to test small fiber function [70–72]. TTT uses a thermode with a baseline temperature of 32C which increases up to 50C or decreases down to 0C [73]. Two methods are available to test for thermal detection and pain thresholds; method of limits (reaction time dependent) and method of levels (reaction time independent). Method of limits starts at the baseline temperature and increases or decreases its temperature. The test-button has to be pressed twice; first when it feels the temperature change and second when the temperature becomes painful. With the method of levels, 2 buttons are available representing yes or no. For each stimulus, the question is asked whether the thermode becomes colder or not [74]. With the method of levels, only thermal detection thresholds are determined and no thermal pain thresholds. According to Table 2, normal temperature detection thresholds lay above 41C and below 25C and temperature pain thresholds lay above 45C and below 5C [15]. In order to exclude large fiber involvement, nerve conduction studies (NCS) are recommended instead of the other QST test procedures [75]. QST improved pain quantification over pain questionnaires and opened new frontiers, beyond NCS capabilities. However, QST also has some limitations. It consists of a set of psychophysical instruments, which requires an alert and focused patient. Also, numerous factors may influence the results, like environmental conditions (seat position, room temperature, illumination and noise level), gender of the tester, instructions provided to the subjects, habituation, cognitive capacity and motor performance [68]. Moreover, lack of age- and gender-controlled normative values has limited routine use. Worldwide uniformity in procedures might overcome most of its limitations [76].

#### Microneurography

Microneurography measures conduction velocity and other properties of small nerve fibers. A needle with a diameter of 1–5μm is inserted into a peripheral small nerve fiber [77, 78]. Microneurography is the only technique which quantifies the sympathetic nerve activity directly [79, 80]. It is often used to measure muscle sympathetic nerve activity and skin sympathetic nerve activity [81]. Some limitations include time-consuming, expensive, requires expertise and strict collaboration with the patient to perform [78, 81]. Therefore, it is not used in routine clinical practice.

#### Nociceptive evoked potentials

With nociceptive evoked potentials, a brief noxious stimuli is applied at the skin to evoke a time-locked response in electroencephalography (EEG) signal [18]. Amplitude and latency are outcome measures from the response in the EEG signal. A*δ* and C-fibers can be examined separate from each other due to difference in conduction velocity. Noxious stimuli can be applied by the use of a laser, contact heat or intra-epidermal electrical stimulation. When small nerve fibers are damaged, conduction velocities will decrease, resulting into increased evoked potential latency and decreased amplitude [82].

### Autonomic function tests

In order to diagnose autonomic peripheral neuropathy, parasympathetic tests (cardiovagal), sympathetic adrenergic tests (cardiovagal), parasympathetic/sympathetic reflex tests (bladder function & pupillometry), sympathetic cholinergic tests (sudomotor) and sympathetic noradrenergic tests are distinguished [83].

#### Cardiovagal tests

Cardiovascular autonomic neuropathy (CAN) often remains undiagnosed and is described in diabetic patients [84]. CAN is divided into three categories 1) possible or early CAN confirmed with one abnormal cardiovagal test, 2) definite CAN with at least two abnormal cardiovagal tests and 3) severe CAN with orthostatic hypotension in addition to definite CAN [85]. The Ewan battery of CAN tests consists of 6 tests 1) heart-rate (HR) response to a Valsalva maneuver, 2) HR response to postural change, 3) HR response to deep breathing, 4) blood pressure (BP) response to a Valsalva maneuver 5) BP response to postural change and 6) BP response to sustained handgrip [86]. Recommendations for the use of cardiovascular tests in diagnosing diabetic autonomic neuropathy include the use of the Ewan test, except for the BP response to sustained handgrip [85]. Other cardiovagal tests include 7) cold pressor and mental stress test and MIBG-scintigraphy.

#### Parasympathetic test

*HR response to a valsalva maneuver:* The Valsalva maneuver consists of voluntary forced expiratory effort against closed airways (for example blowing to the volar side of your hand). Increased thoracic pressure results into a decreased preload, provoking a complex autonomic reflex to compensate for arterial pressure loss. Heart activity is measured with an electrocardiogram (ECG) and RR-intervals are used to determine HR-variability. The maneuver consists of 5 different phases; phase (0) deep inspiration, phase (I) onset of strain, phase (II) continued strain, phase (III) release, phase (IV) recovery [87]. The Valsalva ratio is calculated by dividing the shortest RR-interval in phase II, with the longest in phase IV [88]. Individuals who suffer from SFN, show a lack of bradycardia reflex during phase IV and show a decreased Valsalva ratio [89].

*HR response to postural change:* Changing from supine to upright position results in movement of blood volume from the central- to the peripheral com-partment. An abrupt increase in heart rate is important to maintain homeostasis. The sympathetic system, parasympathetic system and baroreflex together re-main homeostasis. The “30:15” ratio assesses the HR response to postural change, by dividing the bra-dycardia after approximately 30 s with the increased HR after approximately 15 s after postural change. In healthy individuals, HR increases with 10 beats/minute. When autonomic failure occurs due to SFN, there will be no bradycardia [90].

*HR response to deep respiration:* In order to measure the HR variability with deep respiration, the amplitude of individual heart beats on an ECG is most commonly used as measure. Mean square successive difference, mean circular resultant, standard deviation of the RR-interval and expiratory-inspiratory ratio can be used as additional measures. The HR variability assesses the vagus nerve function and is confounded by respiratory frequency and tidal volume, age, hypocapnia and increased sympathetic flow [84, 88]. The vagus nerve is an autonomic nerve fiber and its function might be impaired due to SFN.

#### Sympathetic adrenergic tests

*BP response to a valsalva maneuver:* During the Valsalva maneuver, increased HR occurs in response to decreased BP. The baroreflex response is responsible for the compensatory bradycardia. Patients with autonomic dysfunction show absent overshoot in BP and bradycardia reflex [89].

*BP response to postural change:* Sympathetic ne-rve function can be tested by measuring the BP response to postural change. Redistribution of blood volume, results into a compensatory tachycardia. In healthy individuals BP increases with approximately 10 mmHg. 1-2 minutes after postural change, BP starts to decrease. In patients with severe autonomic dysfunction, BP abnormalities are seen until 5–10 minutes after postural change [88–90].

*BP response to sustained handgrip:* Sustained mu-scle contraction results in a reflex rise in BP. During the sustained handgrip, the subject holds a dynamometer for 3–5 min. BP is measured every minute. The difference between diastolic BP just before contraction and just before release of handgrip, is used as measure of BP response. Patients with autonomic dysfunction, show absent rise in BP. Although this is a valuable research tool, many confounders are responsible for a low sensitivity and specificity. Confounders include poor standardization of muscle effort, reduced muscle afferent activity in trained muscles and reduced muscle chemoreceptor afferent activity due to decreased metabolite accumulation [86, 88].

*Cold pressor and mental stress test:* Immersing one hand into ice water results into increased BP. This reflex is linearly related with increase in muscle sympathetic nerve activity and venous plasma norepinephrine. Mental stress is created by subtracting seven series from 100 or by applying the Stroop color word-naming test. Mental stress also increases the sympathetic outflow. During sympathetic dysfunction, BP increase is lowered or absent [90]. This test is not part of the Ewing test [86], or mentioned in the recommendations for the use of cardiovascular tests in diagnosing diabetic autonomic neuropathy [85]. Sensitivity and specificity are low and inter-subject variability is high [88].

### Combined parasympathetic and sympathetic adrenergic tests

#### Pupillometry

Pupillometry is the study of changes in pupil diameter as function of cognitive arousal [91]. The pupil light reflex is mediated by both, sympathetic and parasympathetic autonomic nerve fibers. This reflex controls the pupil radius as response to environmental light. The parasympathetic system stimulates dilation of the pupil (mydriasis) and the sympathetic system stimulates constriction of the pupil (miosis). The pupil reflex serves as measure for arousal and emotional responses [92], sensory pain [93] and an index for mental load [94]. Selective parasympathetic denervation results in relative mydriasis in light. Selective sympathetic denervation results in relative miosis in darkness and diminution of the startle reflex as seen in Horner’s syndrome [95]. Therefore, the size of the pupil can be used as indicator for several autonomic neuropathies [96]. However, changes of pupil diameter due to arousal or cognitive load, will not exceed 0.5 mm, [91] while switching from light to dark can result into an increase from 1.5 to 9 mm.

#### Bladder function test

Bladder function depends on learned behavior and is under voluntary control in contrast to other visceral organs. Voluntary control is possible due to complex interactions between autonomic and somatic efferent pathways. Afferent components of the sensory input from the bladder neck and urethra consist of A*δ* and C-fibers [97, 98]. The A*δ* fibers are thought to be the primary functional nerves during normal micturition. In contradiction, the C fibers are responsive for pathologic or noxious stimuli, such as chemical irritation or cooling [99, 100]. Bladder dysfunction may develop as consequence of SFN [101, 102]. Bladder function tests consist of cystometry, uroflowmetry, sphincter electromyography and urethral pressure profilometry.

*Cystometry:* Cystometry is usually used to test the passive filling component of the bladder. It evaluates sensation, capacity and involuntary detrusor activity. During the test, bladder pressure is measured via the urethral catheter (P_ves_) and abdominal pressure is measured with an intrarectal catheter (P_abd_). The difference between P_ves_ and P_abd_ is the detrusor or bladder pressure (P_det_) and represents true intravesical pressure readings. With cystometry, an empty bladder is usually filled with sterile water or normal saline. While increasing the volume, the bladder is able to maintain approximately the same amount of P_ves_, also known as compliance [103]. The patient has to mark 3 phases of filling. 1) First sensation of filling. 2) First desire to void. 3) Strong desire to void. Any bladder contraction during filling phase is abnormal. In normal conditions, all three phases will be noticed. An impaired bladder, misses one or more phases and can be related with autonomic dysfunction [104, 105].

*Uroflowmetry:* Uroflowmetry is the analysis of the flow pattern during micturition, the voided volume and residual volume. The uroflowmeter is used to measure urinary stream in milliliters per second (mL/s). Additionally, it measures the voided volume. The residual volume is measured with an Ultrasound (US) scan [106]. A normal flowpattern is continuous with good flow velocity. Decreased flow velocity with increased duration of micturition is indicative for obstruction. An intermitted flow can be indicative for impaired bladder contractility, obstruction or voiding with abdominal straining [107]. A neurogenic bladder often misses the first sensation of filling around 100–200 mL. Normally, discomfort occurs at a filling volume of around 300–500 mL. However, patients with a neurogenic bladder can increase their capacity up to 2L. In neurogenic bladders, 2 patterns do present. The first shows a decreased peak flow and the second shows a prolonged intermitted flow pattern, with the need of abdominal straining to void [89].

*Sphincter electromyography:* With sphincter electromyography (EMG), an electrode is placed in or near the sphincter muscle. Aside from some neurologic conditions, external anal sphincter EMG is the same as external urethral sphincter EMG. Normal voiding starts with relaxation of the sphincter, followed by contraction of the detrusor. EMG shows a slowly increasing activity, until the command to void. During voiding, no activity is measured. After voiding, a constant activity is measured. Several suprasacral spinal cord pathologies may cause detrusor external sphincter dyssynergia (DESD). DESD can result in huge EMG changes as the detrusor contracts involuntary against a relatively closed sphincter. This will result in high pressures and eventually may cause impaired bladder compliance. If no neurologic damage is present, the dyssynergia is behavioral [107].

*Urethral pressure profilometry:* Urethral pressure profilometry draws a pressure profile along the length of the urethra. A catheter with a pressure sensor is inserted in the urethra. The profile is measured during withdrawal of the sensor. The fluid pressure needed to just open a closed urethra is defined as the urethral pressure.

### Sympathetic cholinergic tests

Sympathetic cholinergic tests are based on direct sweat response, after stimulation of M3 muscarinic receptors. Stimulation is achieved by iontophoresis, with thermal, electrical or mechanical stimuli. Me-thacholine, acetylcholine or pilocarpine are pharmacological substances used for iontophoresis of cholinergic agonist. Ionthophoresis stimulates the sweat glands in two ways. Binding to the M3 muscarin receptors on sweat glands results in a direct response of the corresponding sweat gland due to an impulse in orthodromic direction. However, acetylcholine also binds to nicotinergic receptors on the terminal nerve fibers, resulting in an indirect sweat response due to an impulse in antidromic direction [88].

#### Thermoregulatory sweat testing (TST)

TST is a unique technique that provides assessment of preganglionic, postganglionic and central nerve pathways. The patient is positioned nude in a special constructed sweat cabinet, for 40–60 minutes. The sweat cabinet maintains an environmental temperature of 43–46C and a relative humidity between 35–40%[89]. This way, skin temperature is maintained between 38.5–39.5C and should not exceed 40C. A skin temperature above 40C may cause skin injury, confounding somatosympathetic reflex sweating and hydromeiosis (reduced sweat rating at high levels of skin moisture and high temperature) [89]. The patient is covered with an indicator that changes color in the presence of moisture. Color change of the indicator is photographed and processed into a sweat density map generated on standard anatomical drawings. The outcome measure of TST is the percentage of anhidrosis at the anterior body. In healthy subjects, sweat distribution is equal over the whole anterior body. Abnormal sweat distributions can result in distal, segmental, regional, mixed, focal or global anhidrosis [55, 89].

#### Quantitative Sudomotor Axon Reflex Testing (QSART)

QSART assesses the indirect axon reflex mediated sweat response over time. A sweat capsule is used to apply acetylcholine and to measure the humidity caused by increased sweat production [55, 108]. Latency, duration and magnitude of the sweat res-ponse are determined with real-time measurements. Healthy individuals start to produce sweat in appr-oximately 1-2 minutes after the exposure to acetylcholine. Sweat production peaks approximately after 5 minutes and decreases after 10 minutes. Mean sweat output is 2-3μl/cm^2^ for males and 0.25–1.2μl/cm^2^ for females [108]. Sweat output can increase, decrease or persist (“hung up” response). Persistent sweating often relates with hyperalgesia [89]. Advantage of QSART is its temporal resolution. Limitations of QSART are disability of measuring preganglionic lesions, it requires special equipment and is rather expensive [55].

#### Silicone impressions

With silicone impressions, direct sweat response is measured. Sweat glands are stimulated by iontophoresis, for example with the same module used for QSART. After 10 minutes, the capsule is removed, the skin is dried and a silicone mold is applied. The silicone mold is liquid and cures after approximately 5–10 minutes. Sweat droplets leave imprints in the silicone mold. Number of droplets, droplet area and droplet volume are used as measure for small nerve fiber function [55, 109]. Healthy individuals have 311±38 sweat droplets/cm^2^ on hands and 281±38 droplets/cm^2^ on feet. Abnormal impressions are predominantly seen in patients with anhidrosis or hyperhidrosis [55]. Anhidrosis might be caused due to SFN, since sympathetic cholinergic nerve function is innervated by small nerve fibers. However, many other disorders are associated with anhidrosis. Moreover, this method lacks temporal resolution and is therefore disabled to determine latency and duration of the response.

#### Quantitative direct and indirect reflex testing (QDIRT / acetylcholine Sweat-spot Test)

QDIRT is developed in order to combine the advantages of QSART and the silicone imprint technique. Advances in photography are sufficient to enable quantification of dyed sweat droplets like the silicone imprint technique. Iontophoresis of acetylcholine is applied in combination with an indicator dye. Sudomotor function is determined with temporal resolution in the same way as QSART, while spatial resolution (droplet size and number) is determined similar to the silicone imprint technique [110]. Autonomic function has limitations as it is influenced by body temperature, humidity, hydration status, nicotine and room temperature [108, 110]. An important additional limitation is near absent response in female subjects. Female subjects show low sweat volumes, in combination with rapid evaporation in cool dry air [110].

#### Sympathetic skin response (SSR)

SSR measures changes in skin potentials. The sources of skin potentials are sweat glands and epidermis. Multiple stimuli have been used to elicit SSR, but most of those show no precise onset or indefinable strength of the stimuli. The best definable stimulus is an electrical square wave pulse [89]. The stimulus disturbs the autonomic nervous system, generating a change in skin potential. When the autonomic nervous system is affected by SFN, SSR latency will be delayed. The skin potential is measured with standard EMG electrodes. Presence or absence of the response as well as amplitude and latency are reported from the SSR. Absence of SSR might be caused by habituation or an inefficient stimulus [55]. Latency is hard to determine for multiple stimuli, and amplitude varies. Advantages of SSR are easy performance, it requires little additional training, no special equipment next to standard EMG, it is believed to be useful, clinically meaningful, reliable and has extensive published data [89].

#### Sudoscan/EZSCAN

Sudoscan measures the electrochemical skin conductance (ESC). Two nickel electrodes are placed on the subject’s hands and feet. The electrodes alternate between cathode and anode. The anode supplies a low DC-current, which results in a voltage (<4V) to the cathode. Consequently, a current starts to run, with the amplitude related to the concentration of chloride ions. The concentration of chloride ions depends on the height of the applied current and influences the ESC. ESC is determined from the derivative current associated with corresponding applied voltage, which is expressed in microSiemens (μS). Since patients with SFN show anhidrosis, lower concentration of chloride ions results into decreased conductance. As a result, patients with impaired small fiber function, show lower ESC to the corresponding voltage [56].

#### Laser doppler imaging flare (LDIflare)

LDIflare assesses the indirect axon reflex mediated blood flow on the foot. The subject acclimates in a room of 25±1 C. With the use of a 0.64 cm^2^ skin heater, the skin on the foot is heated up to 44C. Within 20 minutes, maximal hyperemia is achieved and can be assessed with the use of laser Doppler imaging (LDI). An area of 3.5 cm^2^ surrounding the heated skin is scanned to measure the axon reflex mediated blood flow. The axon reflex mediated blood flow is an autonomic function innervated by small nerve fibers. The LDIflare area is calculated with software and expressed in cm^2^. LDImax is the mean flux in the area of the skin heater. LDIflare and LDImax are expressed in arbitrary perfusion units [111]. LDIflare is reduced in diabetic patients compared to healthy controls and correlates with NFD [112].

#### Neuropad

Neuropad is a bandage containing Cobalt II compound. Sweat production causes Cobalt II compound to change from blue to pink. The time required to change the color of all Cobalt compound is a measure of sudomotor function. Sudomotor dysfunction is reported when the required time interval of at least one foot, is above 10 minutes. Advantages of the Neuropad are fast results, its non-invasive nature, simplicity and a high sensitivity [113, 114]. Furthermore, it can be used to stage [115] and predict [116] neuropathy.

#### Skin wrinkling

Water-induced skin wrinkling (WISW) is a reliable sympathetic nerve function test. One hand of the subject is placed in 40C water for 30 minutes. In order to assess skin wrinkling, both hands are compared with each other. The wrinkle can be subdivided into five grades, with grade 0, no wrinkling and grade 4, wrinkling completely distorting the fingertip [117]. Warm water induces vasoconstriction resulting in a negative digit pulp pressure. The pressure gradient between superficial and deep skin structures, results in a downward pull of the overlying skin. Due to the internal structures, skin folding presents [118]. Sympathetic nerve fiber dysfunction prevents vasoconstriction. As a result, skin wrinkling remains absent after exposure to warm water [118].

### Sympathetic noradrenergic test

#### Quantitative pilomotor axon-reflex test (QPART)

The pilomotor axon-reflex test is an unique axon-reflex test, since it is activated by the noradrenergic sympathetic nerves. Iontophoresis with phenylephrine results in local direct and indirect piloerection. The line connecting the most peripheral edges of erected hairs is the outline of the total area. The indirect area can be calculated by subtracting the area of phenylephrine application from the outline area. A local topographic map is created with the use of silicone impressions. Limited literature is available to determine whether this test is able to diagnose SFN [119, 120].

#### MIBG/SPECT

Imaging cardiac sympathetic function can be used to determine SFN [123]. I-meta-iodobenzylguadine (MIBG) is radioactive and can be visualized with the use of single photon emission computed tomography (SPECT) [123]. I-MIBG acts as a substrate for norepinephrine and thus marks post-ganglionic sympathetic noradrenergic innervation. The heart-to-mediastinal uptake ratio (H/M ratio) and washout ratio (WR) are used to quantify sympathetic innervation [90, 121]. Autonomic or cardiac disorders result in either impaired uptake of I-MIGB or accelerated washout after 3–5 hours [121]. CAN is visualized in amongst others diabetics and Parkinson’s disease (PD) patients. A high correlation between H/M ratio and IENFD is shown in PD patients [121], but not in diabetes [122].

### Non-small fiber nerve tests

#### Peripheral nerve ultrasound

Recently, US measurements of the sural nerve revealed structural change of the sural nerve in subjects with SFN [123]. The superficial peroneal nerve is also assessed, but shows no change between SFN and healthy controls. Cross-sectional area significantly increases in patients with SFN from 2.7±0.6 mm^2^ in healthy volunteers to 3.2±0.8 mm^2^ in subjects with SFN. Thickness-to-width ratio does not show difference between healthy volunteers and subjects with SFN [123]. The exact pathophysiology of enlargement of a large fiber like the sural nerve, is unknown. Theories include loss or injury of distal small fibers or impaired sodium channel function resulting in impaired axoplasmatic flow. As consequence, axons degenerate and swell [29].

#### Functional magnetic resonance imaging (fMRI)

Functional activity in the brain can modulate the perception of pain. Balance between non-nocicepti-ve and nociceptive information, control nociceptive information transmission in higher centers. Multiple studies assessed brain functionality in subjects with SFN and healthy controls. Subjects with SFN show volume reduction in pain-processing regions (anterior cingulate cortex). Moreover, the degree of volume reduction correlates with the degree of IENFD decrease [124]. Figure 2 shows an overview of the available diagnostic tools for testing pure SFN and their corresponding measuring area. Most methods only measure post-ganglionic NFD or function. Two methods which do test SFN, but are not based on small nerve fiber testing, are DNA analysis from blood samples and US of the peripheral sural nerve.

### AUC values

**Fig. 5:**
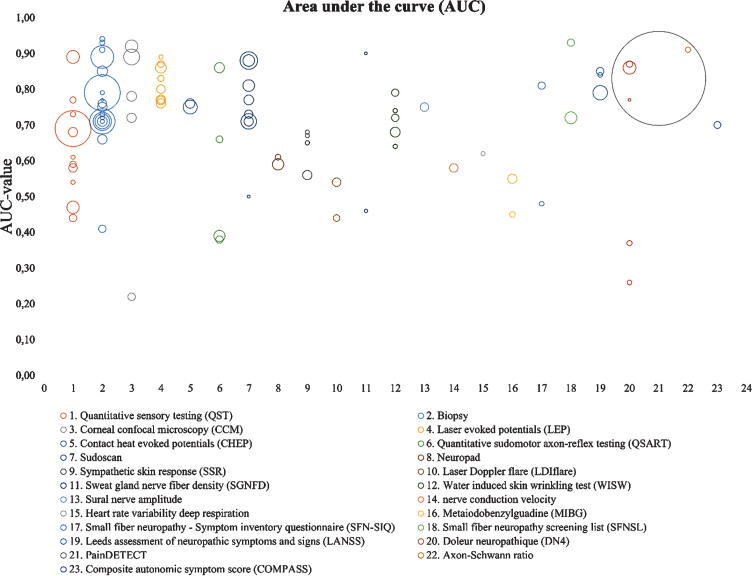
AUC overview of several diagnostic methods. It determines the diagnostic accuracy of each method. The size of each bubble, represents the study size. Unmyelinated axon-Schwann ration is calculated based on biopsies and not further reviewed in this article.

Figure 5 shows AUC values for all techniques published in the last 19 years. The AUC value represents the direct relation between sensitivity and specificity. Each bubble represents one article and the diameter represents the sample size (n). This visual representation gives insight on the number of studies investigating different techniques and show the spread of diagnostic accuracy. From these figures, however, it cannot be concluded which technique shows the best performance. The large range of AUC values within one method, might be caused by multiple parameters as outcome measures, with some measures more sensitive compared to the other.

### Clinical applicability

Not all methods are ready for routine use. Table 4 sums up the limitations for each method and their clinical applicability level. The classification is designed for this review and based on the Dutch healthcare system, which consists of 4 hospital levels.

• Level 1: Clinical applicable in general hospitals
• Level 2: Clinical applicable in top clinical hospitals
• Level 3: Clinical applicable in academic hospitals
• Level 4: Only used in research setting or not yet applicable for routine use

Each technique is labeled based on current applicability.

**Table 4:** Level of applicability and limitations for each method

Method	Limitations	Level of applicability
	*Quantification of small nerve fiber density*
Skin biopsy (IENFD)	–Thickness of tissue sections not applicable for routine diagnostics	Level 3
	–Fluorescence microscopy less available
	–Counting IENF number per epidermal length in light microscopy cannot easily be standardized without computer-assisted image analysis [146]
	–Morphological changes also appear in healthy subjects [54]
	–Morphological changes may occur without decreased IENFD [147]
	–Decreased IENFD is not correlated with pain intensity in all cases [148]
	–Normal IENFD + morphological changes may appear in an early stage of SFN and show decreased IENFD after repeated biopsy [54]
	–May be unclear in patchy diseases [131, 149]
	–The pattern of symmetric, non-length dependent neuropathic pain with face and trunk involvement suggests a selective disorder of dorsal ganglia cells sub serving small nerve fibers. [150]
	–Training required [151]
Sweat Gland Nerve	–Complex 3D structure	Level 4
Fiber Density (SGNFD)	–No standardized and validated method available [151]
	–Limited depth of view Biopsy thickness of 50μm
	–Labor intensive (30–40 hours only for staining)
	–Not applicable for routine use [55]
Corneal Confocal	–Limited availability	Level 4
Microscopy (CCM)	–Not yet approved by the FDA for clinical use [63]
	*Sensory function tests*
Quantitative Sensory	–Great inter-observer variability	Level 2
Testing (QST)	–Lack of world-wide standardization [70]
	–Test results may be influenced by patients (lack of) motivation
	–Interpretation of normative values is difficult due to great differences in methods between normative value studies
	–The comparison between the painful and contralateral site is difficult due to the absence of the minimum meaningful difference [152]
Microneurography	–Invasive	Level 4
	–Technically demanding
	–Labor intensive
	–Measurement of a single small nerve fiber [153]
	–No normative values available
	–Only used in studies [154]
	*Parasympathetic tests and sympathetic adrenergic tests*
Cardiovagal tests	–Response is dependent on duration, strain level, rate of pressure change, body position in which it will be performed (not standardized), fluid status, duration and position of rest before the maneuver, rat of inspiration, time of the day, room temperature, food intake, caffeine, nicotine, medication,, manner of starting (voluntary or after signal), and age. [87]	Level 1
	–Complex underlying physiologic mechanisms [86]
	–Lack of widespread standardization in methods and normative values
	–Many different devices available, including custom-made
	–Need for training and expertise [85]
Pupillometry	–Magnitude of reflex is variable and affected by the initial pupil size	Level 1
	–Without solid standardization of testing conditions, the test lacks sensitivity [89]
	–Difficult to interpret the results, disorders do present symmetric in the eyes. [95]
Bladder function tests	–Good calibration is required	Level 1
	–Good cooperation with and instruction of the patients is necessary
	–Quality check of the results is important, may be influenced by artefacts [106]
	*Sympathetic cholinergic tests*
Thermoregulatory Sweat	–Not applicable for routine use, except in highly specialized centers	Level 3
Test (TST)	–Time-consuming
	–Special equipment is required
	–Requires a lot of space
	–Requires special preparation of the patient [55]
Quantitative Sudomotor	–Special equipment is required	Level 2
Axon-Reflex Test (QSART)	–Staff needs to be trained
	–Expensive [55]
Silicone impressions	–Prone to artifacts	Level 4
	–Suboptimal quality of dental molds [55]
	–Time consuming
	–No temporal resolution
	–It cannot discriminate between neurogenic and gland-related impairment
	–Not standardized
	–Challenging skin and environment preparation [155]
Quantitative Direct and	–Staff needs to be trained	Level 4
Indirect Test of sudomotor	–If the dye on a skin area is already color-changed, it cannot be quantified
function (QDIRT)	–Low inter-individual comparability due to lack of predefined skin areas
	–Indirect areas may lack indicator dye
	–Not yet validated [155]
Sympathetic Skin	–Low inter-individual comparability	Level 1 [156]
Response (SSR)	–It declines with age and may be absent in subject older than 50 years
	–It is a surrogate measure of sudomotor function, patients with congenital
	absence of sweat will still have an SSR response [55]
Sudoscan/EZSCAN	–Normative values used in studies are inconsistent	Level 1 [56]
	–Insufficient evidence support the claim that sudoscan tests sudomotor or sensory nerve fiber function
	–Many studies are manufacturer-supported [157]
Laser Doppler Imaging	–The original method is time consuming (30–90 min),	Level 4
flare (LDIflare)	which limits routine clinical use
	–A modified method is proposed to be less time consuming (<30 min) [158], but is not yet widely applied by other research groups [159]
Neuropad	–Data on diagnostic value differ [113, 160, 161]	Level 1
	–Manufacturer’s instructions, might be suboptimal
	–Limited data available on skin temperature influences [161]
Water Induced Skin	–No standard method to determine abnormal threshold	Level 1
Wrinkling (WISW)	Each laboratory should determine own age-matched cut-off values for normal and abnormal wrinkling [162]
	*Sympathetic noradrenergic tests*
Quantitative Pilomotor	–Only tested in small populations	Level 4
Axon-Reflex	–No normative values
Test (QPART)	–Influenced by strong emotions and room temperature [155]
MIBG/SPECT	–No standardized method available	Level 2
	–No normative values
	*Non-small fiber tests*
Peripheral UltraSound (US)	–Only one suboptimal study is performed to show potential of this method	Level 4
Functional Magnetic	–Only one suboptimal study is performed to show potential of this method	Level 4
Resonance Imaging (fMRI)	–No standardized method specific for SFN diagnosis is available [124]

These levels are based on the Dutch health care system. Level 1: Applicable in general hospitals (easy applicable in any hospital) Level 2: Applicable in top clinical hospitals (not applicable in small hospitals, but also not limited for academic hospitals). Level 3: Only applicable in academic hospitals Level 4: Not yet applicable for clinical routine use, only used for research purposes.

### Diagnostic work-flow

**Fig. 6:**
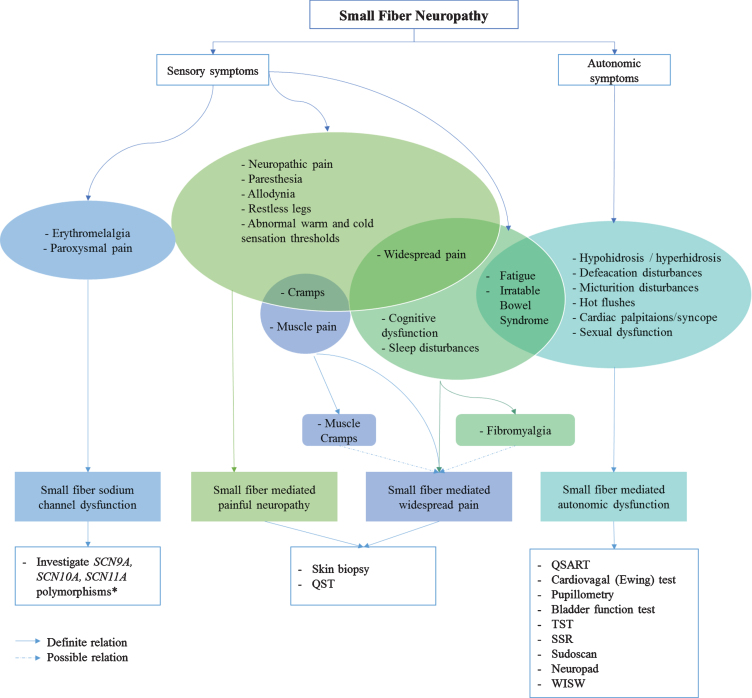
Roadmap; from symptoms to phenotype, from phenotype to diagnostic method. * Many variants are probably nonpathogenic or variant of undetermined significance (VUS). *Abbreviations:* QSART, quantitative sensory axon reflex test; TST, thermoregulatory sweat test; SSR, sympathetic skin response; WISW, water induced skin wrinkling.

Since small nerve fibers are part of both the somatic and autonomic nervous system, the location of the neuropathy influences the type of symptoms. Therefore phenotyping SFN into “small fiber sodium channel dysfunction”, “small fiber mediated painful neuropathy”, “small fiber mediated widespread pain” and “small fiber mediated autonomic dysfunction”, has been proposed [12]. Although symptoms may overlap between different phenotypes, SFN patients may differ from each other and require different therapies. For example, sodium-channel blockers are only effective in patients with proven sodium-channel mutations [12]. Figure 6 shows a roadmap proposing sufficient diagnostic methods, based on different phenotypes. Only methods currently available for routine use are suggested (i.e. level 1–3).

## DISCUSSION

In order to present an overview of the AUC values, all available diagnostic methods are included. However, due to the large amount of methods, no subdivision is made for multiple parameters within one method. For example, TTT and CCM measurements both result into 4 different outcome measures. Each test shows different sensitivity and specificity values.

In order to show an overview of all methods as complete as possible, no studies are excluded based on sample size. For many techniques, sensitivity and specificity are only calculated based on small sample sizes. Since the available methods to test for SFN are so numerous, it would add little information if sensitivity and specificity were only included for a few methods, based on large samples sizes. As a consequence, these values are less reliable, compared to studies with large sample size. In addition, positive and negative predictive values are not presented due to limited data.

SFN is associated with many different underlying etiologies. Since pathophysiology and cause are un-known, SFN etiology may differ between different populations. In case of different etiologies, some methods can be more or less sensitive for specific phenotypes.

Sensitivity and specificity depends on the amount of patients with SFN included in the study. When fewer patients with SFN are included in the study, sensitivity and specificity are less reliable compared to studies including a large amount of SFN patients. No criteria are used to exclude studies with lower number of SFN patients compared to control groups.

Clinical applicability levels are designed for this review, based on the Dutch healthcare system. A level is ascribed based on research papers and reviews, which describe the limitations of a technique and whether it is used for research or clinical use. In case of clinical use, an estimation is made about how simple the method can be implemented. Labor intensity, expenses and the required size of research room are amongst others factors which influence the clinical applicability levels.

The diagnostic workflow presented in Fig. 6, is based on the results of extensive literature research. It has not yet been validated and consensus for a definite SFN diagnosis, is still lacking.

## CONCLUSION

Diagnosing SFN remains a challenge. While small fibers have a wide range of functions, current diagnostic tools only focus on specific areas. Therefore, SFN in areas outside of the diagnostic test range might be missed. Moreover, tools focusing on different areas do not correlate with each other. A reliable test that examines all parts of the small fiber system has yet to be developed. In the absence of a true gold standard, the most reliable diagnosis of SFN in daily clinical practice is made using a combination of tests based on structural *and* sensory function tests of the small fiber nerves. Ideally, Na-channel mutation research and autonomic tests should be added to also test all phenotypes of SFN. A diagnostic work-flow based on phenotypes is suggested. Given the magnitude of the clinical problem further research is necessary in order to obtain a simple, reliable and non-invasive technique to assess SFN.

## CONFLICTS OF INTEREST STATEMENT

The authors declare no competing financial interests

## DISCLOSURE OF FUNDING SOURCES

ZonMW-TopZorg St Antonius Care grant 842002003

## MEETINGS

This paper has been presented at the NRS Young Investigators Symposium, 15 November 2019, Amsterdam
